# Advancements in Root Growth Measurement Technologies and Observation Capabilities for Container-Grown Plants

**DOI:** 10.3390/plants4030369

**Published:** 2015-07-03

**Authors:** Lesley A. Judd, Brian E. Jackson, William C. Fonteno

**Affiliations:** Department of Horticultural Science, North Carolina State University, Campus Box 7609, Raleigh, NC 27695, USA; E-Mails: brian_jackson@ncsu.edu (B.E.J.); wcf@ncsu.edu (W.C.F.)

**Keywords:** container production, rhizometrics, rhizosphere, root systems

## Abstract

The study, characterization, observation, and quantification of plant root growth and root systems (Rhizometrics) has been and remains an important area of research in all disciplines of plant science. In the horticultural industry, a large portion of the crops grown annually are grown in pot culture. Root growth is a critical component in overall plant performance during production in containers, and therefore it is important to understand the factors that influence and/or possible enhance it. Quantifying root growth has varied over the last several decades with each method of quantification changing in its reliability of measurement and variation among the results. Methods such as root drawings, pin boards, rhizotrons, and minirhizotrons initiated the aptitude to measure roots with field crops, and have been expanded to container-grown plants. However, many of the published research methods are monotonous and time-consuming. More recently, computer programs have increased in use as technology advances and measuring characteristics of root growth becomes easier. These programs are instrumental in analyzing various root growth characteristics, from root diameter and length of individual roots to branching angle and topological depth of the root architecture. This review delves into the expanding technologies involved with expertly measuring root growth of plants in containers, and the advantages and disadvantages that remain.

## 1. Introduction

Approximately 90% of the horticultural plants grown in the $16 billion United States greenhouse, nursery and floriculture industries are produced in containers [1]. These U.S. and other global industries produce a variety of crops including bedding plants, foliage plants, potted flowering plants, potted nursery stock, and other floriculture/nursery crops, all grown in a myriad of container sizes. Container production of ornamental horticulture crops has increased substantially over the last several decades, due to several advantages container production has over traditional field production. Plants grown in plastic containers have been shown to have a greater fine root mass compared to field-grown plants [2,3]. After transplanting, these plants were better able to meet transpiration needs with minimal roots loss compared to harvested field-grown plants. Container-grown plants have more fine roots along the outside of the root ball, which come into contact with the backfill soil when transplanted [3].

Root growth of crops grown in containers is a central element in overall plant performance, whether it is during propagation, production or post-production (e.g., transplant success) due to the combined functions of roots being anchorage, support, and water and nutrient uptake [4]. Container design can also have an effect on root growth, as it can influence the morphological and physiological characteristics of seedlings/plugs/liners [5]. Plants grown in smooth-sided plastic containers for a long production cycle can result in deformed roots or underdeveloped root systems because lateral roots cannot extend horizontally, as they would in the landscape, and therefore the roots grow vertically to the bottom of the container and begin to circle the container’s edge [6]. This can lead to problems in the landscape, such as plant instability, reduced shoot growth, overall plant decline, and mortality [6]. Container depth is considered an important variable influencing plant and root morphology as it is directly related to water holding capacity, humidity, and air ventilation [5,7,8].

Considering the large portion of the horticultural industry involved with growing plants in containers and the importance of understanding the physiology and morphology of roots, the factors that influence root growth in container production need to be investigated as well as their effect on root systems. Rhizometrics is a term derived from *rhizo*- (rhizosphere) and -*metrics* (series of parameters or measures of quantitative assessment used for measuring, comparisons or tracking performance or production) to describe several methods either developed or examined by researchers at North Carolina State University (Raleigh, NC, USA) to observe and quantify root growth of plants in containers [9]. However, this term can be used to describe all root zone measurements, including field studies. The need to examine new techniques of observing and measuring root growth of plants, especially plants grown in containers with soilless substrates during production, arose from the observed disadvantages noted with current and past methods.

Root growth and root architecture are frequently excluded in horticultural research [10], and the study of natural root development is a challenge due to the difficulty of root observations in containers during crop production [11]. Strategies and techniques for observing, studying, and quantifying root growth have been reported over the past nine decades. Many of the known methods of measuring root systems are tedious and time-consuming, and the accuracy of their results is generally low and very few studies use standard methods [12]. Moreover, plants produced in containers are subjected to more frequent applications of water, fertilizer, plant growth regulators and pesticides than field-grown plants. These increased inputs and restricted soil/substrate volumes increases rates of plant growth and flowering over plants grown in the field. Root development in these containers is also different but somewhat more poorly studied than many field-grown plants. Fortunately, many advances have been made in recent years for the study of root measurements; including techniques that can be easier, faster, reproducible, and more descriptive of root growth, such as plant image analysis software. While many of these techniques apply to field systems, they are particularly useful in studying container-grown plants.

The objectives of this paper are: (1) to review the many methods of measuring root systems, both past and present; and (2) to highlight some important modern techniques for measurement and analysis of root system characteristics of plants produced in containers.

## 2. Root Anatomy and Morphology

The diversity of root systems can cause links between certain root properties and their functions to be uncertain [13]. In order to obtain a full picture of the root system, multiple parameters will need to be measured [13]. In many situations, only rough estimates of root presence or function may be needed; however much of the work done with plant roots has been to advance our knowledge on the interrelationships of root measurement with function to create important root parameters to measure [13]. Several key root system parameters include: root length, root weight, root volume, root:shoot ratio, specific root length, branching pattern, horizontal distribution, root hair density, root uptake ability, root hydraulic conductance, and root viability [13]. These root traits can predict important root functions within and among species, including respiration, water and nutrient acquisition, lifespan, and decomposition. However, our understanding of many of the linkages between root form and function is still in its infancy [14].

### 2.1. Root Hairs

As early as the seventeenth century, researchers began to realize that plants absorbed water rather than soil and that nutrient substances were absorbed by the roots at the level of the root hairs [15,16,17]. Typical root hairs have a cylindrical, straight form with a dome-shaped tip, that often form a right angle with the main root surface. They appear a short distance behind the root tip where the cells are dividing, and persist for a relatively short time depending on the plant species [18]. As the hairs die off, new ones are formed closer to the root tip; a process termed the migration of root hair zones [18]. Root hairs have been considered an important component of plant roots, and many researchers have included qualitative and quantitative reports of root hairs with overall root system function. In 1949, Dittmer [19] investigated root hair variations between different families of angiosperms and noted that considerable variation was found in the diameters, lengths, shape, and color between the different species, but within any one species the size and color was relatively constant. He [19] reported that root hairs were present in many root systems of different plants and were important enough to attempt to measure. Living root hairs were scattered over the entire surface of all the roots of a winter rye plant (*Secale cereal* L.) and the surface area of these root hairs was nearly twice that of the main, larger roots [20].

### 2.2. Root Types

Another important root characteristic that has been linked with mechanical support and acquisition of water and nutrients is root architecture/types and diameter. These traits can be used to predict yield under specific conditions, such as drought or low fertility, and understanding the diversity and development of root architectural traits is crucial as it is indicative of plant fitness [21]. For root types, three classes of roots usually described are: the taproot, lateral roots, and adventitious (shoot-borne) roots [22]. Cannon [23] was the first to attempt to classify root systems, by striving to recognize characteristics that were constant or similar between genetically similar plants growing in varied habitats. Other authors have reported that there is a fourth root class, termed basal roots [22,24,25]. The taproot is the first root to emerge from the seed, and is considered the primary root while the basal and lateral roots that develop from the taproot are considered to be secondary roots [22,25]. These secondary roots then in turn produce tertiary, quaternary and further roots [26,27]. The types of roots in root systems can be used to characterize the plant and environment; for example if the root system consists of a taproot with little basal or lateral roots, it is considered an early root system on a young plant for most species [25]. The structure of the root organ itself is very consistent between different species; but the number, placement and direction of growth of each root in the system is highly variable, even among genetically similar plants [27]. The apical regions of roots allow plants to adapt their morphology and organ development to the encountered environmental conditions [28]. The number and length of secondary roots varies greatly depending on the plant species, soil composition and water and nutrient availability [27].

### 2.3. Root Architecture

Root architecture refers to the spatial configuration of the entire root system; however studies of root architecture usually do not include fine structural details, such as root hairs [29]. Root architecture is generally quite complex, and is different from morphology, root topology (branching) and distribution (amount/presence of roots in a positional gradient) [29,30]. Root architecture can include topography and distribution, and these descriptions are usually easier to measure. The architecture of a root system can determine its exploration of spatial domains in the soil, as well as its ability to respond to possibly localized available nutrients in the soil/substrate [26]. However, little is known about root architecture and its roles for the plant because it is difficult to observe, quantify, and interpret without destroying the native architecture [29].

### 2.4. Root Diameter

Root diameter varies both within and between species and can sometimes be used to describe what the root and plant experience in the surrounding environment. Root diameter determines the length of root that the plant can produce for unit input of resources to the system [26]. The diameter of fine roots in forests have a strong role in determining the fine root turnover; as diameter increases, the root turnover decreased [31,32]. Peat and Fitter [33] report that it is often found that the roots of species that form mycorrhizal associations, especially obligate mycorrhizal species, have much coarser roots (larger diameter) and no, or very few, root hairs compared to species with no mycorrhizal associations that have very fine roots and copious amounts of root hairs. Some plant species produce fine roots when grown at a low nutrient supply [26]. The diameter of roots also seems to be a good predictor of the effect of mechanical impedance and soil/substrate pore size, because data obtained by Wiersum [34] demonstrates in a greenhouse study that a root is only able to penetrate a pore which has a diameter exceeding that of a young root and Goss [35] reported results that mechanical impedance caused the plant to grow superficial and densely branched root system where the roots did not grow past eight centimeters of depth. Roots are often larger in diameter than the water-filled pores of soil at field capacity (*i.e.*, pores with diameter ˂60 µm), and so pores that drain freely are the main spaces in which roots can grow [36]. In the field, it was demonstrated that roots can force their way through pores smaller in diameter [37], and the root tip must exert sufficient force to deform the soil [36].

## 3. Methods for Measuring Root Growth

The study of root growth began in the field over nine decades ago with agronomists who studied root growth in various soils. According to Weaver *et al*. [38], an exact knowledge of root development of crop plants, their position, extent and activity, is of paramount importance to a scientific understanding of plant production. Methods of measuring root growth and specific root characteristics have ranged widely in technique and/or equipment needed (Table 1), and several are dependent on conditions such as field-grown or container-grown plants.

### 3.1. Field-Grown Methods

Several techniques used to measure root growth in the field included trenches, photography and excavation [38]. McDougall [39] used the horizontal glass-plate method, where a square foot of glass was buried five cm below the surface of the soil and covered with felt roofing so it could be removed to count the number of roots growing against the glass. McDougall [39] also used the vertical glass-plate method, where holes were dug into the earth two and a half feet wide by five feet long and two feet deep, and a glass plate was placed against one side of the hole and the entire hole was covered with a board cover to block out light. Other techniques described by Schuurman and Goedewaagen [40] include monoliths, soil cores, and profile walls. More recent work has been done with rhizotrons, minirhizotrons or transparent walls/windows [41].

**Table 1:** Overview of most frequently used methods to measure or to analyze root systems, and selected studies reporting or using them. (Adapted from Reubens *et al*. [12]).

Method	Author(s)	Information Type	Destructive to roots?	Advantages (+)/Disadvantages (−)
**Field methods**					
Photographs or drawings	[38,42]	Qualitative analysis, 2D root morphology	No	(+) Copy of the exact root structure visible, easy and rapid (photographs) (−) tedious (drawings), blurry (photographs), no statistical inference or quantitative information, only qualitative commentaries, 2D only, problems with root overlap
Trench/window	[40,42]	2D spatial root distribution	Yes/No	(+) easy to record root data, repeated measurements on specific roots (−) static, limited 2D area, roots and structure could be destroyed by digging process, aberrant root growth along installed window
Pinboards/monoliths	[40,42,43,44]	Length, weight, diameter, distribution pattern	Yes	(+) view some natural arrangement of roots (−) requires some skill, labor-intensive, large losses of fine roots
Auger/core	[40,41,42,45]	Length, weight, diameter, distribution pattern	No	(+) easy (−) requires large number of samples, labor-intensive, sampling depth limited, time-consuming processing in lab
Rhizotron/minirhizotron/mesorhizotron	[46,47]	Dynamic 2D information on root morphology, growth and turnover	No	(+) repeated measurements on specific roots (−) expensive, possibly labor intensive (construction and analyzing data), aberrant root growth along window
Above-ground rhizotrons	[11,48,49]	Dynamic 2D information on root morphology, growth and turnover	No	(+) repeated measurements on specific roots (−) aberrant root growth along window
**Container methods**				
Root washing	[50,51,52,53]	Root dry weight, shoot:root ratio, diameter, distribution pattern	Yes	(+) whole root system visible (−) large losses of fine roots, loss of natural positions/architecture, time-consuming, tedious
Root rating	[54,55,56,57]	Root density, appearance, branching and distribution pattern	No	(+) easy, rapid (−) subjective measurement, qualitative, human error
Transparent containers/substrates	[58,59,60,61,62,63,64]	Root density, appearance, branching and distribution pattern	No	(+) whole root system visible, 3D, more natural architecture (−) different environment compared to soils and soilless substrates
Horhizotron™	[10,55,65]	Root density, appearance, branching and distribution pattern	No	(+) repeated measurements on specific roots, lightweight materials used (−) only for large plant use—starting with 3.78–11.35 L root balls, materials not permanent/fixed, easily breakable, aberrant root growth along window
Mini-Horhizotron, rhizometer, hydraulic conductance flow meter	[9,66,67,68]	Root density, appearance, branching and distribution pattern	No	(+) repeated measurements on specific roots, lightweight materials used, materials permanent, hard to break (−) only for small plant use—seeds/plugs/liners, aberrant root growth along window
**Digital imaging**				
Image Analyzing Computer	[69]	Branching and distribution pattern	Yes	(+) less time-consuming, less subjective (human) (−) harvested roots, only photographing small sections of roots at a time, problems with root overlap
WinRHIZO, RootReader	[70,71,72,73,74,75]	Root density, angles, appearance, branching and distribution pattern, root length, root surface area	Yes/No	(+) easy, rapid, less subjective (human), greater range of measurements (−) may only work on washed roots (destructive), problems with root overlap
NMR and X-ray CT	[76,77,78,79,80,81]	Root length, growth, volume repartition	No	(+) report image of whole root system (−) far from being practical, roots grown in small containers only

#### 3.1.1. Trench, Photographs and Drawings

Methods of measuring roots has varied over the years, and many past methods that were thought of as illustrative during that time are now limited and no longer used. Weaver *et al*. [38] used the same excavating method that was commonly used in 1922, digging a trench along the side of the plant at a depth of five feet and of a convenient width. In some cases, it was possible to view the entire root system and the usual practice was completed. The usual practice of this time consisted of writing a working description, noting variation and hand-drawing a replication of the root system on a large drawing-sheet to the exact measurements [38]. In other cases, the roots were destroyed by digging and had to be reconstructed. Photographs were often found to be blurry, not allowing the viewer to perceive the finer roots; therefore hand-drawn pictures were the best representative of the root system [38]. The most extensive and comprehensive root excavations since the classical work of Weaver has been done by Lore Kutschera in Austria [42]. Kutschera has investigated a wide range of grasses, herbaceous plants and agricultural crops in Europe, and she proposed the idea of digging trenches on the south side of plants to allow the investigator to shade the exposed roots with their body and this also prevented the glare of the sun shining directly into the investigator’s eyes [42]. Digging trenches or installing root windows is utilized as a technique to this day as a way to record root images in situ. However, the trench/window remains static and represents a limited, two-dimensional area which does not provide information on the total root system extension [58].

#### 3.1.2. Pinboards and Monoliths

Monoliths, or pinboards, can be used both with field-grown and with container-grown plants. The pinboard method is thought to give a fairly complete depiction of the structure and shape of the root system. The pinboards can be constructed from 1–1.5 cm thick plywood with holes drilled 5 cm apart both horizontally and vertically that hold pins [40]. In the field, a pit is dug against the plant, the dug-out wall smoothed and the pinboard may be placed and pressed against this wall and then a steel cable may be passed down each side of the pinboard in a sawing movement so that the soil surrounding the pinboard is cut away and the pinboard is free to pull out with the soil and roots still held by the pins [40]. The specimen can now be transported to a laboratory where the soil will be washed off, leaving the roots arranged around the pins in a similar architecture found in nature [40]. Kono *et al*. [43] and Kano-Nakata *et al*. [44] used a “root box-pinboard” method to quantitatively and qualitatively measure root system morphology. The root box was made of transparent solid vinyl chloride with dimensions of 25 cm length, 2 cm width and 40 cm depth, making a relatively small box due to all the handling required to place the pinboard on it at the day of harvest [43]. Both authors found this method was an easy and effective way to view the natural morphology of the root systems, only requiring around fifteen minutes per sample per person to harvest [43,44]. However, there were several disadvantages; Kono *et al*. [43] reported the size of the box was limiting for growth, and three different types of dyes had to be used to get an optimum contrast among the root system members.

#### 3.1.3. Auger and Cores

Another method, core sampling, can be used to measure root establishment in the field or landscape after transplanting. A soil core auger is used to extract sample soil-root cores from the landscape in order to separate the roots from the soil. From these extracted roots, root growth can be expressed as weight, surface area, volume, diameter, length or the number or root tips, as well as root length density that is determined by the root length per unit soil volume [42,82]. Core sampling has advantages over only taking a portion of the root system compared to harvesting the entire plant, and the equipment required for sampling and for root separation is inexpensive compared to other methods such as deep pit digging. Researchers also drilled holes into the field soil in order to place polyvinyl chloride (PVC) tubes that would later be removed from the field so that the roots growing within could be extracted from the soil by washing and then observed/measured [45].

#### 3.1.4. Rhizotron, Minirhizotron and Mesorhizotron

Another common technique currently used today is rhizotrons. The first rhizotron, designed by Rogers [83] in 1933, was constructed in East Malling, Kent, England from 1960–1961 at the East Malling Research Station, which is famous for its development of dwarfing rootstocks of fruit trees. The word rhizotron is coined from Greek words *rhizos* for root and *tron* for instrument and can be defined as a facility or building designed underground for viewing and measuring plant roots and underground structures through transparent surfaces that may be in contact with the natural soil [46]. It is a tool for making nondestructive, repeated measurements of root systems at a large field-scale. Rhizotrons are one of the earliest non-destructive techniques for observing root growth in soil, and they have several advantages and limitations [84]. Advantages include the ability to take successive measurements on the same individual root and to rapidly see the length increases [84]. Sensors and cameras can be installed to measure soil conditions and record time-lapse photography. Roots growing along the transparent wall can be traced as the roots grow, to provide information on speed of root growth and root density [85]. However, the primary disadvantage of the rhizotron is its expense of construction and operation [84]. A rhizotron constructed at Auburn, Alabama in 1969 cost about $40,000 and during the thirteen years of operation added to this cost by $50–100,000, spent on instruments, control systems, and updated computer systems [86]. Current costs of constructing a rhizotron would be substantially greater [84]. Huck and Taylor [86] discuss several disadvantages of rhizotrons; the finite number of repetitions, the immobility of the structure and changing of the soil environment when the rhizotron is installed. Also, the viewing surface of the rhizotron may not be representative of the roots in the bulk soil at depth and after research is conducted, the soil might need to be replaced, in which case the replacement soil may have altered populations of worms, fungi, bacteria and insects compared to the native profile [46].

A similar technique to the rhizotrons is the minirhizotron, originally proposed by Bates [87] using a mirror and a battery-operated lamp mounted on the end of a stick to see roots intersecting a glass tube in the ground. Throughout later years, this minrhizotron was improved by others to create the modern minirhizotron that uses a color video camera with a right-angle viewing attachment that can be lowered into the underground tube, and images can be recorded on video or photographs taken, both of which have improved quality images due to the modern technology [41,84,47,88]. One of the greatest limitations of the minirhizotron is the number of tubes required to accurately estimate rooting [84]. It is suggested to use a minimum of eight tubes in a single plot and it requires 30 to 45 minutes to install each tube [84]. Another disadvantage of the minirhizotron is the amount of labor/time required to collect the pictures from every tube and analyze them [89].

#### 3.1.5. Above-Ground Rhizotrons

**Figure 1:**
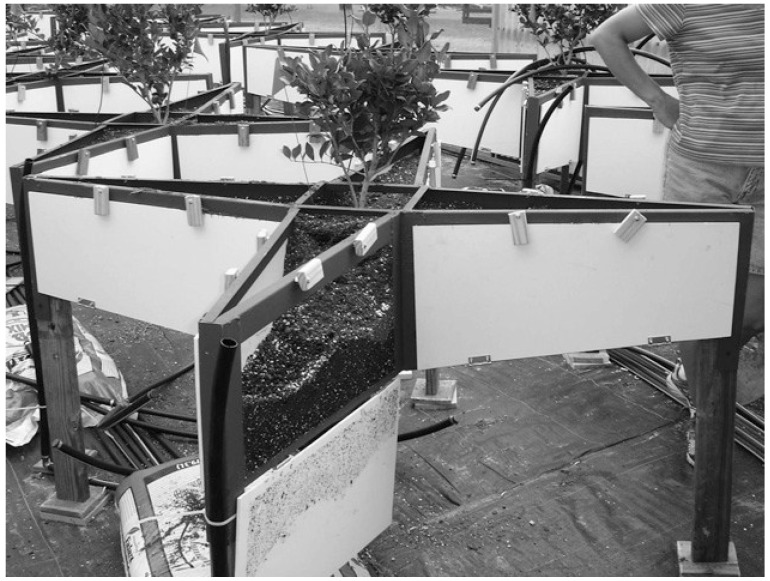
A large-volume rhizotron with a transplanted one-year-old *Ligustrum japonicum*; the window is open for demonstration of viewable rhizosphere (original picture by Silva and Beeson [11]).

In 1985, James *et al*. [48] suggested a new nondestructive root measurement technique similar to the rhizotron, which eliminated the issues of expense and requirement for specialized equipment. James *et al*. [48] still called their apparatus a rhizotron or mini-rhizotron, and it was constructed of two 20 cm × 20 cm × 0.5 cm transparent plexiglas plates held one cm apart by plastic tubing. This rhizotron could be used in or out of a greenhouse, shaded by panels or aluminum foil, inexpensive, and created a small box to view growing roots in. Neufeld *et al*. [49] created a root box similar to James *et al*. [48] that was slightly larger and grew plant roots between a plexiglass sheet and a nylon sheet with soil medium on the other side of the nylon so complete view of the roots could be had. Pan *et al*. [90] developed a new portable rhizotron system called mesorhizotron, to observe root growth in different cropping systems, soil conditions and environments. The mesorhizotron has a transparent face on a box that is buried in the soil, and a portable hand scanner can be placed into the box to scan the transparent wall view [90]. Supporting hardware for the scanner and software for storing and analyzing the images were also required for the mesorhizotron, and each box required five vertical scans [90]. Silva and Beeson [11] developed a large-volume rhizotron for aboveground observation of undisturbed, natural root growth of woody plants, as seen in Figure 1. The large-volume rhizotron was to mimic in-ground conditions, including enhanced drainage for evaluating effects of soil moisture deficits on root growth [11].

### 3.2. Container-Grown Methods

The importance of root system development in relation to plant growth is often overlooked due to the roots growing into the substrate and not directly visible/observed. Measuring root systems in containers decreases the area/substrate volume needed to find all the roots of the one plant, and allows for a more complete excavation of the root system compared to plants grown in the field [58]. In viewing the whole plant, the shoots and roots are in constant competition for energy and nutrients for their development. A measure of the resultant pattern of differential growth, expressed as the shoot:root ratio, provides an index for the performance of each organ in a certain growth environment [50]. These shoot:root ratios may help ascertain how environmental and chemical factors affect and modify the growth of the shoot and root [50]. Shoot: root ratios are often measured with the destructive method of comparing dry weights of roots and shoots. Media must be washed away from the roots and then the roots are placed in an oven at 70 °C for several days, until all water has evaporated.

#### 3.2.1. Root Washing

Using this method, much of the fine roots and root hairs are lost in the process, as well as the natural positions and arrangements of the roots. In standard methods of washing and storage of root samples, losses of dry weight from 20% to 40% may occur [51,52], and could therefore be defined as destructive. Very fine roots are difficult to wash and even by using a sieve with a mesh size of 0.2 mm^2^, these roots still may be lost [42]. Washing substrate or soil from roots can be very time-consuming, with each sample taking anywhere from 3 to 25 min [53].

Benjamin and Nielsen [53] developed a root washer that has the capability to accommodate large samples for washing. However, this root washer is still destructive and some information, such as root length, root diameter classes and root weights, is not obtained with this method. Substrate can also be removed from the roots using compressed air in order to view the root system and collect dry weight measurements [5]. Extracting roots from soil or soilless substrates is a process included in many root measuring techniques, even though the primary disadvantage of any method using root washing or removal would be the potentially for great root loss. After removing the soil or substrates from a root system, the root growth potential can be measured using a volume-displacement technique involving suspending roots in a clear graduated cylinder to observe the volume displaced and therefore report the root system volume [91].

#### 3.2.2. Root Rating

Root rating can be a simple and easy way to qualitatively describe root balls of container-grown plants, washed roots and propagative rooted cuttings. Ratings can evaluate root density, appearance, branching and distribution [54,55]. Subjective root ratings can be done on root balls, where the roots are observed growing on the outside of the substrate or by destructively washing the roots and rating the uncovered root system [56,57]. Root ratings can also be measured with the rhizotrons, minirhizotrons and Horhizotron™ by estimating the root density observed through the transparent walls [55]. Walters and Wehner [57] found root rating was a simple and accurate method for determining cucumber root growth in the greenhouse. The authors note that that rating is a subjective measurement, and the person rating must first understand how to accurately rate the size of the root system [57]. The rater must determine beforehand the categories of the rating scale, and can even lay out some root systems that refer to one of the categories, to be consulted to when a judgment is made [57]. Another method that required careful washing and soil removal from seedling root systems is a photoelectric device termed the rhizometer. This device can be used to estimate root surface areas of seedlings by using a light source on which the root system is placed and a photocell to measure the reduction in light due to the roots and a galvanometer which in turn measures the decrease in output from the photocell [92]. This is a rapid method of measurement, however sources of error may occur if roots cross one another and this would lead to underestimates of the root area, as well as the possibility of actively growing roots being translucent and therefore not give the same light reduction compared to opaque roots [92]. This method also measures one root system measurement: root surface area. The natural architecture of the roots was lost once removed from the natural growing environment and other complex and interesting root measurements were lost as well.

#### 3.2.3. Transparent Containers and Substrates

Plant root system architecture exhibits the adaptability and dynamic force of plant roots, as root system architecture responds to the environment in order to optimize acquisition of important soil or soilless resources. Visualization of natural root systems of plants grown in situ (*i.e.*, in containers) is often obscured when plants are grown in soil or soilless substrates, and roots can form extensive networks in these substrates which inhibits their easy removal for observation. Therefore, several laboratory and greenhouse approaches have been conceived to overcome these limitations. To access the root system, plants can be grown in clear pots to view roots emerging against the edge. Windows can be cut out of containers to allow for the observation of root growth in those areas [59]. Plants can be grown in rhizoboxes constructed out of clear PVC boxes to view root development along the edges [58]. However the challenge of visualizing the whole root system remains due to the opaque nature of substrate/soil particles. To facilitate viewable access of the whole root system, plants can be grown in liquid culture, on the surface of agar or paper, or clear gel substrate in transparent containers [60,61]. Root growth and morphology can be easily monitored without interference in hydroponic or in aeroponic culture systems [58]. Transparent gel substrates have the advantage of providing a solid rhizosphere for the growing plant roots, which allows the roots to grow in three dimensions (3D) for complex root measurements [61,62]. Fang *et al*. [63] found that using transparent containers and gel substrate to measure plant roots was very effective when capturing in situ 3D root architecture images without any contact or perturbation of the plant root system. However, the growth of plants roots in transparent substrates may not be similar to how they would actually grow in soils or soilless substrates; research has found that plants grown in phytagel (*i.e.*, agar substitute composed of glucuronic acid, rhamnose and glucose; Sigma-Aldrich Co., St. Louis, MO, USA) had greater lateral root lengths and reduced lateral root density compared to plants grown in soil [64]. This has led to using “transparent soil” in order to mimic physical and chemical properties of natural soil systems; Nafion is a transparent ionomer that has properties similar to vermiculite and sand and has been shown that plants grown in transparent soil had lateral root lengths and densities more similar to root growth of plants in soil [64].

#### 3.2.4. Horhizotron™

The smaller, above-ground rhizotrons, minirhizotrons and root boxes are still currently used, and adaptions to these techniques have created new designs, such as the Horhizotron™. The Horhizotron™ is a non-destructive technique used to measure lateral root growth from an original root ball of a container-grown plant, allowing for post-transplant assessment [10]. The center of the Horhizotron™ fits a range of 3.78–11.35 L (1–3 gallons) size root balls. Plants are placed in the center with eight panes of glass that extend away from the root ball in a four-pointed star shape [10]. The substrate in each quadrant can be modified in various ways in order to examine the effects of different rhizosphere conditions [10]. Each quadrant can be filled with a different substrate [55], or the quadrants can be divided with one type of substrate on the lower half and a different substrate on the upper half [65]. The Horhizotron™ can easily be used in a greenhouse or in the field, due to the lightweight materials used and ease of assembly. The disadvantages of the materials used are; the glass panels are not permanently placed and can move and crack, the shade box does not restrict all light from the root system, and the limitation of only being able to use large container plants to observe root growth.

#### 3.2.5. Mini-Horhizotron

**Figure 2:**
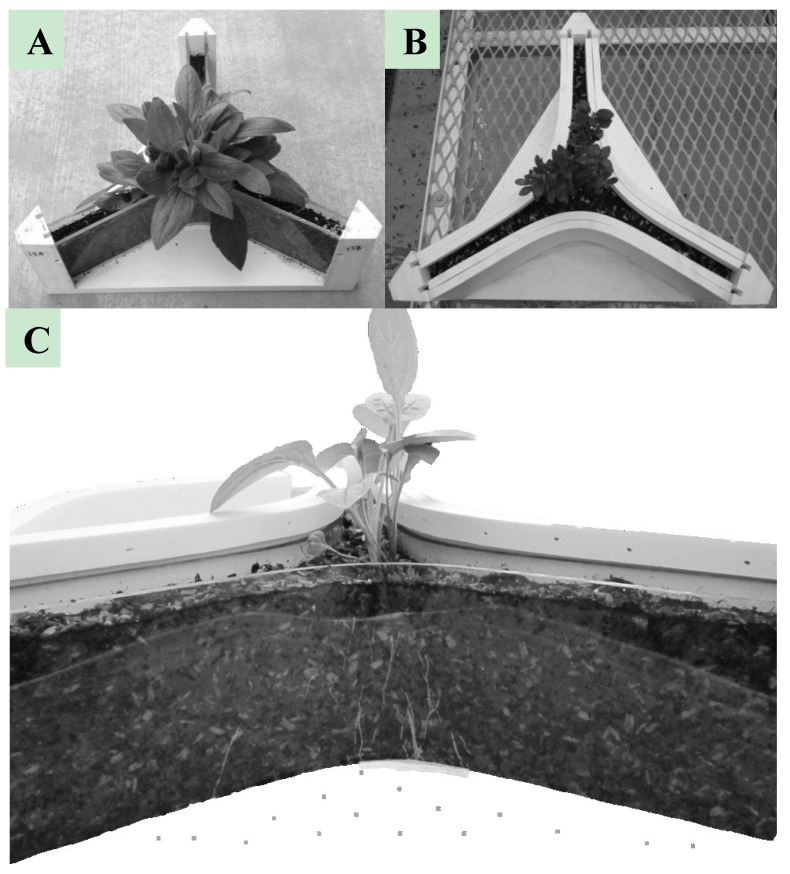
(**A**) Mini-Horhizotron with a *Rudbeckia hirta* plant, transplanted from a plug, with all shade panels removed; (**B**) All shade panels in place on the mini-Horhizotron, blocking out sunlight from the *Ilex crenata* rhizosphere; (**C**) Side view of the mini-Horhizotron with visible *Rudbeckia* roots growing in the substrate.

The mini-Horhizotron was developed to study root growth of seeds, liners and plugs after planted or transplanted into common greenhouse containers (*i.e.*, 10–16.5 cm diameter pots) under production conditions. The design of the mini-Horhizotron is comprised of a three-chamber configuration suitable for observing and measuring root growth by utilizing the clear walls as demonstrated in Figure 2. The mini-Horhizotrons have a substrate volume similar to a standard greenhouse container, and the height of the mini-Horhizotron (10.2 cm) is also similar to a 16.5 cm diameter container (11.8 cm), providing similar air and water profiles comparatively [66]. However, the surface area of the mini-Horhizotron is almost three times larger than a container, allowing for an increase in potential viewing of roots as they explored the substrate [66]. Previous work has shown that plants grown in the mini-Horhizotron had similar dry root masses when compared to plants grown in a greenhouse container, supporting using the mini-Horhizotron to observe and measure effects during production conditions [66]. In order to block sunlight from the rhizosphere, shade panels slide directly against the clear walls (Figure 2B). This design allows for repeated measurements of roots from a plug/liner/seed as they would fill out a standard greenhouse container; and this method aids in better understanding of root growth patterns, problems and potential [9]. Each chamber of the mini-Horhizotron has a width of 2.5 cm to maximize the chance of roots growing against the clear walls [66]. Possible root measurements in situ include; root length, speed of root growth, presence and quantity of root hairs, and root branching/architecture [66]. The mini-Horhizotrons have been used to measure root growth and potential effects of substrate type on root growth during greenhouse plant production. The effect of different rhizosphere conditions (e.g., different substrate or fertilizer treatments) can also be observed and quantified, as well as monitoring disease pressures [67]. The mini-Horhizotron is lightweight and portable, allowing for its use as an observational tool for use in education classrooms with observations of plant physiology and plant pathology. Root measurements were easily accomplished without the destruction of the root system or substrate removal, and root tracings and/or photographs could be used to measure root surface area/density with software programs.

#### 3.2.6. Rhizometer

**Figure 3:**
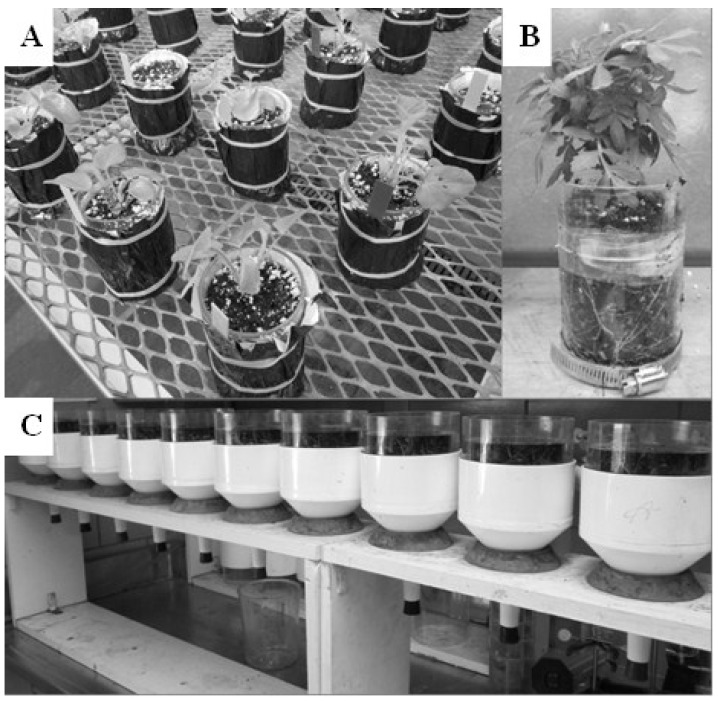
(**A**) Rhizometers covered with foil to prevent sunlight from reaching the rhizosphere are grown on a greenhouse bench; (**B**) Foil removed to view roots visible along the clear cylinder of the rhizometer; (**C**) Harvested rhizometers with shoots removed, in the North Carolina State University Porometer method [93] to measure substrate physical properties.

Another apparatus developed to observe and measure root growth and also its effect on container substrate physical properties over time: the rhizometer. For this device, multiple measurements are taken into consideration; measurements of plant roots as well as measuring the changes in substrate physical properties (*i.e.*, air space, container capacity, and total porosity) as the roots continue to explore the substrate over time [9]. Substrate physical properties can be measured by placing a rhizometer with shoots removed into the North Carolina State University Porometer method [93]. Rhizometers are comprised of clear cylinders that allow for both viewing a growing root system and also in situ measurements including; root length, speed of root growth, presence and quantity of root hairs, and root branching/architecture (Figure 3). Roots are also easily traced when grown in the rhizometer, allowing for software program to analyze the root system. Judd *et al*. [68] found that the measured total root length from tracings of several plants grown in rhizometers were highly correlated to the dry mass of the root system, therefore potentially reducing the need to destructively wash and extract roots from the substrate.

#### 3.2.7. Hydraulic Conductance Flow Meter

The other traits have been investigated for correlation to root growth, such as the physiological trait of root hydraulic conductance, which can be an indicator of plant performance and adaptability to a given environment. The Hydraulic Conductance Flow Meter (HCFM; Dynamax, Inc., Houston, TX, USA) is an apparatus that can measure both root and shoot hydraulic conductance with minimal disturbance to the root system, although the measurement is destructive to the plant as a whole, due to the shoot being excised from the rootstock. The shoot stem or rootstock is fitted with water filled tubing of the HCFM and once connected, the HCFM uses consistently increasing pressure to cause water to flow into the root or shoot system [9]. The pressure versus the water flow measurement is used to estimate the hydraulic conductance. Using the HCFM to measure root hydraulic conductance, it has the potential to correlate with root mass and therefore possibly reduce the need to destructively wash and extract roots from the substrate.

### 3.3. Digital Imaging

Rhizotrons, minirhizotrons and other transparent wall/container designs commonly have the option to use digital imaging to measure root systems. Digital imaging includes photographs or videos of the transparent walls, scanned images of roots floating in water, or scanned drawings of root tracings. Photographs or videos can be taken when using the minirhizotron method, in order to examine and measure root lengths and density using a computer or recording equipment. Ottman and Timm [69] discuss measuring roots by harvesting them from the root clumps of onion plants, photographing the root segments, and using the photographic negatives in the Image Analyzing Computer to predict root length or area. The advantages of using photoelectronics methods to quantify roots are: being less time-consuming and less dependent on human judgement than other methods. Root systems can be imaged directly with a digital camera when grown in glass containers and/or transparent substrate such as gellan gum or transparent soil replacements [21,64]. As technology advances, photographs or scanned images can be used by computer programs to evaluate several root measurements, as images have several key features that make them valuable for plant research. Even digital images are more advanced and can contain several layers of information, making them a viable option as a tool for root measurements [94,95]. The resolution of digital imaging combined with the objectivity of automated analysis allows for more repeatable analysis of broad and productive sets of measurements [60]. There are numerous computer programs, both commercially and freely available, and there are nineteen commonly used and known computer programs [94]. Several of these programs include RootLM, RootReader 2D, EZ-Rhizo, WinRHIZO and WinRHIZO Tron. Some of these programs have the capability to measure complex root system architecture traits, such as branching, density, angles, total area and root order [61]. With the advantages of using computerized programs for measuring specific root characteristics, increasing amounts of published research using plant image analysis software have been detected [94]. All of these applications have their strengths and weaknesses, and for the entire root system some morphological traits can be analyzed. These include total root length, root average diameter, total root surface, root tips, and root volume [95].

#### 3.3.1. WinRHIZO, RootReader

RhizometOne software program that has been used in scientific literature is the WinRHIZO (Regents Instruments, Quebec City, Canada) program. WinRHIZO is based on an optical scanner instead of a video camera, because scanners produce high-quality images [70]. Possible measurements for the WinRHIZO system are; total length, projected area, surface area, root tips, branching points, and root length for different width intervals chosen by the user [70]. According to Fang *et al*. [71], WinRHIZO is relatively inexpensive and suitable for both large and small-scale experiments. Villordon *et al*. [72] used WinRHIZO to classify lateral root growth of sweetpotato (*Ipomoea batatas* “Beauregard”). Sweetpotato roots were washed, placed in water and scanned to be analyzed by WinRHIZO [72]. Debris, such as sand particles and broken root segements, in the washed roots were removed manually in the WinRHIZO program [72]. Root type classification was based on predetermined diameter intervals designed by the researchers, and used to show different root stages [72]. While the scanner allows for clearer images, the roots must be washed and the substrate removed, and measurements of the root system over time cannot be observed with this program. Washing roots can cause a loss of fine roots and disturb the natural architecture of the root system. Unless the roots are floated in water, as done by Villordon *et al*. [72], the natural architecture is gone. Another program WinRHIZO Tron, developed by the same company, is used to analyze images from rhizotrons, minirhizotrons or other transparent wall techniques. Root tracings can also be scanned into the WinRHIZO Tron program, and root length can be converted to root mass [73]. However, the images to be used with WinRHIZO Tron are often unfocused and blurry, so the user has to manually select the roots on the image and trace the length for the program to know what to measure. Other programs are available that also used images from scanners; Benjamin and Nielson [53] used a flat-bed scanner to digitize images of roots and used the commercially-available image analysis software Sigma-Scan™ to determine surface area of the roots; scanners can also be used to acquire images of seedling roots growing along an agar surface, aeroponically grown systems and rhizotrons [60].

Other available programs can use digital images from cameras or scanners to measure root systems. ROOTEDGE is a software program developed to measure areas, perimeters, lengths and widths of digitized roots using a scanner and an algorithm for the measurement calculations [74]. The program is also capable of performing some basic image-processing operations, however the program assumes that the objects (*i.e.*, roots) in the image will be black and the background will be white [74]. RootReader 2D was developed at Cornell University (Ithaca, NY, USA), and images of intact root systems can be uploaded into the program and root growth responses quantified from whole root systems or specific roots of interest [75]. Several sophisticated image analysis programs have been developed to measure plant root system architecture and traits; these include RootTrace, REGR analysis, Kine-Root, and Root FlowRT. These programs focus on analyzing root growth and architecture from a time series of images and are extremely useful for measuring effects of temperature, genotype, and nutrient availability on root growth [61].

Even with the numerous advantages of using computer software programs to measure root systems, disadvantages can be found as well. Kano-Nakata *et al*. [44] used digital photography and a computer program to measure root length; however they found this program underestimated root length because of overlapping roots, especially fine lateral roots. The Image Analyzer Computer used by Ottman and Timm [69] did not differentiate between viable and nonviable roots or other extraneous organic matter found in the root samplings. Clean, washed roots work well for image analysis, leading to one of the major disadvantages for many computer programs as the roots cannot be completely distinguished separately from background noise, similarly colored substrate particles (e.g., perlite or fertilizer granules) and/or organic matter. In these cases, some software allows the roots in the image to be traced using the computer cursor; however the accuracy of this method depends greatly on hand-eye coordination of the operator [89]. The methods to obtain the images can also be a disadvantage; as the greatest drawback of minirhizotron systems has been the tedious, time-consuming process of translating qualitative information from observations to quantitative data.

#### 3.3.2. X-ray CT and NMRI

Other techniques to measure the 3D root systems are available, and have been researched. X-ray computed tomography (CT) and nuclear magnetic resonance imaging (NMRI), both of which are non-invasive and non-destructive, offer a new approach for the study of undisturbed root growth over time. The CT method uses X-rays to measure the photo-electrical absorptions or scattering to scan the roots growing in soil/substrate contained in PVC tubes and produces a 3D image [76]. The sample is rotated between an X-ray source and a detector, and a series of 2D projections are recorded from which a 3D volume dataset can be reconstructed [77]. With resolution ranging from 10 to 500 μm, the X-ray CT-scanning technique is also a unique tool for 3D visualization and quantification [78]. Recent applications have included lateral root development or root elongation rates. The whole root system in its environment/substrate can be measured allowing for non-destructive measurements, however a major problem is often other structures surrounding the roots, such as water-filled pores, can lead to low contrast hindering straightforward segmentation of the roots from the background [77]. Studies conducted by Tracy *et al*. [79] reported twelve hundred image projections were captured for each planted container measured with CT. The root system models segmented from the CT image data can be used to quantify root length, volume, surface area, mean diameter, root tip diameter and vertical root depth [79]. To acquire high quality images, long scan times are necessary; Daly *et al*. [80] reported scanning a sample for 105 min with 360° rotation and 1440 projection images produced. Using the CT method with high resolution scanners may lead to a wider use of CT in plant sciences [77]. This method can also be utilized beyond plant roots to measure rhizosphere hydraulic properties or to characterize soil aggregate properties.

The NMRI method uses proton signal intensities to measure spatial array and therefore produce an image of the root system [81]. Protons are highly abundant in living tissues and particularly in water molecules. When using NMRI, is it necessary to distinguish protons in roots from protons in soil in order to measure a correct image of the root system; unfortunately most natural soils are unsuitable for NMR imaging [81]. Strong magnetic fields and radio frequency fields produce 3D datasets of samples [77]. The density of protons or their physical and chemical microenvironment can be exploited, to produce a strong difference between “root water signal” and “soil water signal” which provides a very high contrast between roots and their background [77]. Research applications range from phytopathology, storage root internal structures and water mobility in roots [77]. Scanning with the NMRI can take as long as the CT scan, however the CT scan may take longer to produce segmented images [77]. Roots can appear to be much thicker in the NMRI compared to CT, and this is caused by the much coarser spatial resolution of the NMRI [77]. Metzner *et al*. [77] reported that the thinnest roots detected with NMRI were about 250 μm in diameter.

Both CT and NMRI techniques can investigate specific root details. For finely graduated root diameters, CT may be advantageous as it provides higher spatial resolution [77]. For larger pot diameters, NMRI can deliver higher fractions of the root systems than CT, most likely due to the strong root-to-soil contrast achievable by NMRI [77]. Complementary information can be gathered with CT and NMRI, a combination of the two techniques could open a whole range of additional possibilities for the anaylsis of root systems [77].

## 4. Conclusions

Special methods and techniques are required to investigate root systems of plants since they are hidden in the soil or substrate in which they are grown. Discussed in this review are several techniques and the expanding technologies involved with expertly measuring root growth of plants in containers, and the advantages and disadvantages still found today. Traditionally, techniques like coring, trenching, excavating, rhizotrons, pinboards and root washing have been used to access roots in the field, however some of these methods are destructive and repeated observations or measurements cannot be made. Methods of measuring root growth in the field have been expanded with new techniques developed to measure root growth of container-produced plants. These methods, such as the Horhizotron™, mini-Horhizotron, and rhizometer, are useful tools in measuring root characteristics under production conditions of fertilizers, watering or plant growth regulators. The Horhizotron™ investigates post-transplant conditions of large plants while the mini-Horhizotron explores variables found during plant production conditions. Using transparent containers or substrates allows the complete root system to be viewed virtually at once without the tedious and somewhat destructive root washing process. Current methods, such as the mini-Horhizotron, also have great potential for non-destructive measurements of inoculated disease pressures and root loss. Developing computer software for use in measuring roots has improved the depth and range of root characteristics measured. Using these non-destructive methods in real-time appear to be the direction for future techniques in Rhizometrics. This will include more options of non-destructive methods as well as non-invasive methods like the NMRI and X-ray CT. The field of Rhizometrics is enlarging in promising and intriguing directions, providing more root measurement options in both research and education.
